# Supplementary material for: “Maternal Vaccination Greatly Depends on Your Trust in the Healthcare System”: A Qualitative Study on the Acceptability of Maternal Vaccines among Pregnant Women and Healthcare Workers in Barcelona, Spain
Source: Vaccines (Basel). 2022 Nov 25;10(12):2015. doi: 10.3390/vaccines10122015 (PMC9783547; doi:10.3390/vaccines10122015)
Supplement: Supplementary file 1 [file vaccines-10-02015-s001.zip › vaccines-2012772-supplementary.pdf]

vaccines

MDPI

Supplementary Materials:

Annex 1. Interview guide for pregnant women (in Spanish)

GUÍA ENTREVISTA A MUJERES EMBARAZADAS

1. Presentación

· Buenos tardes. Lo primero, presentarme. Soy __, trabajo en el Instituto de Salud Global de Barcelona.

· En primer lugar, agradecerle su disponibilidad para participar en esta entrevista a partir de la que esperamos conocer su opinión y puntos de vista, ya que es importantes para el estudio.

· En esta entrevista vamos a hablar sobre su experiencia del embarazo y sobre su punto de vista sobre la vacunación.

· Este encuentro va a durar aproximadamente sobre media hora. Va a ser grabado con su consentimiento para asegurar que la información no se pierda. Los datos serán tratados de manera anónima y confidencial. En el momento de analizar y publicar los datos, se hablarán de las opiniones generales de todos los participantes del estudio. TODA LA INFORMACIÓN ESTARÁ CODIFICADA.

1.1. Datos sociodemográficos

Primero le voy a preguntar unos datos sociodemográficos que voy a anotar, pero que no voy a grabar. Le voy a avisar en el momento que empiece a grabar.
(DATOS SOCIODEMOGRÁFICOS)

1.2. Introducción

¡Empezamos a grabar!

· Bueno, hoy es __(fecha)__, a las__(hora)__ y esta entrevista está siendo grabada a partir de una llamada telefónica.

· Únicamente voy a recoger su consentimiento para participar de manera oral, así que, puede decir en voz alta si acepta participar en el estudio, y ser grabada.

2. Historia personal

Hábleme un poco sobre usted…

- ¿Cómo se enteró de que estaba embarazada?
- ¿Llevaba mucho tiempo buscando?
- ¿Cómo es su día a día con el embarazo? (condiciones: vida cotidiana)
- ¿Quién la ayuda? ¿Qué apoyos tiene durante el embarazo?
- ¿Cómo se siente con el embarazo?
- ¿Y a nivel de salud? ¿Tiene alguna patología?

- ¿Actualmente está trabajando?
- ¿En qué área? ¿Desde cuándo está en este lugar?
- ¿Tiene estudios en algún ámbito?

3. Percepciones generales de las vacunas
- ¿Qué opinión tiene sobre las vacunas en general? ¿Cuándo oye "vacuna", que le viene a la cabeza?
- ¿Ve igual si se trata de vacunas en personas adultas, mayores, niños/as…?
- (si no lo ha dicho ella) ¿Cree que las vacunas en general funcionan /son eficaces?

4. Percepciones generales de las vacunas en el embarazo
- ¿Y qué opina de las vacunas durante el embarazo?
- ¿Cree que las vacunas son seguras en el embarazo o que tienen algún riesgo?
- (En el caso que crea que haya riesgos) ¿Cuáles?
- (en el caso que no lo haya dicho ella) ¿Son mayores los beneficios o los riesgos? ¿Cree que en general los beneficios de las vacunas superan sus riesgos?
- ¿Cree que otras mujeres embarazadas de su entorno sienten/perciben lo mismo?

5. Conocimientos de vacunas en el embarazo
- ¿Qué sabe acerca de las vacunas en el embarazo? (tipos, administración…)
- ¿En qué momento del embarazo (semanas, trimestre…) se ponen?
- ¿Sabe que enfermedades previenen?
- ¿Para qué se utilizan? ¿Para proteger a la madre, al feto o ambos?
- ¿Cómo afecta a las mujeres embarazadas por el hecho de estar embarazadas?
- ¿Cómo cree que deciden vacunarse las mujeres embarazadas de su entorno o no hacerlo?
- ¿Van a casa y se lo piensan? (o ya lo tienen claro…)
- ¿Cree que suelen confiar en las recomendaciones de los sanitarios?
- ¿Qué opinión tiene de la vacuna de la tos ferina?
- ¿Y de la vacuna de la gripe?
- ¿Y de la vacuna de covid-19?
- ¿Cree que la gente/la sociedad tiene una percepción similar de estas tres vacunas o que hay alguna diferencia?

6. Experiencia en embarazos previos
- ¿Ha tenido otros embarazos anteriormente?
- ¿Ha sido vacunada alguna vez durante el embarazo?
- ¿De qué vacunas?
- ¿Cómo se sintió cuando se vacunó? ¿Tuvo miedo?
- En el caso de que se vacunara…
- (únicamente si ha tenido embarazos anteriores y se ha vacunado) ¿Cree que ha prevenido infecciones en su bebé?
- - En el caso de que no se vacunara…
- ¿Cuál fue el motivo? ¿Sintió miedo de hacerlo?
- ¿Quién tomó la decisión de vacunarse o no?

- ¿Cómo la tomó?
- ¿Con quién lo consultó?
- ¿Dónde se informó?

(Fuentes de información: pareja, matrona, ginecóloga, pediatra de sus otros bebes, familia, amigas, otros medios etc.)

- (Si ha tenido un hijo/a en un país extranjero), ¿conoce el calendario vacunal?

7. Experiencia en el embarazo actual

- ¿Le han propuesto vacunarse?
- ¿Y qué ha decidido? / (si es un embarazo reciente) ¿Y si se lo propusieran?
- ¿Cómo ha tomado esta decisión? / ¿Cómo tomaría esta decisión?
- ¿Cuándo le propusieron vacunarse? ¿tuvo (suficiente) tiempo para pensárselo? ¿Lo pensó mucho? ¿Por qué?
- ¿Cómo se siente/se ha sentido al tomar esta decisión?
- ¿Quién ha sido su mayor apoyo para decidir esto?
- ¿Quién tomó la decisión de vacunarse o no?
- ¿Cómo la tomó?
- ¿Con quién lo consultó?
- ¿Dónde se informó?

(Fuentes de información: pareja, matrona, ginecóloga, pediatra de sus otros bebes, familia, amigas, otros medios etc.)

8. Fuentes de confianza y credibilidad

- ¿Cuáles son sus fuentes más confiables para obtener información de las vacunas en el embarazo?
- ¿Y para decidir vacunarse?
- ¿Cree que la opinión del profesional sanitario puede afectar a su decisión de vacunarse?
- ¿Cuál cree que es el papel de las autoridades sanitarias/médicas para vacunarse/en todo esto de la vacunación?
- ¿Cree que este papel ha cambiado tras la pandemia?
- ¿Confía más o menos en las recomendaciones de vacunas?
- ¿Considera que ha tenido la opción de elegir?
- ¿Se siente que ha sido bien informada?
- ¿Qué le ha gustado de cómo se lo han contado informado y qué no?
- ¿Qué le ofrece confianza?
- ¿Qué vacuna le genera más confianza/credibilidad para vacunarse en el embarazo: tos ferina, gripe, covid y otras?
- ¿Por qué? ¿Y la que menos? ¿Por qué?

9. Ensayo clínico

- ¿Estaría dispuesta a participar en un ensayo clínico de una vacuna?
- ¿Por qué motivos?

- ¿Y en una situación de pandemia?
- ¿Conoce alguna vacuna que esté en ensayos clínicos actualmente en mujeres embarazadas?
- ¿Sabe que actualmente se están haciendo ensayos con estreptococo tipo B y virus respiratorio?
- ¿Participaría en alguno de estos ensayos?
- ¿En qué casos?
- ¿Hay alguna situación en la que cree que participaría?

10. Otros temas

- ¿Hay algún tema que no hayamos tratado y que quiera comentar o algo sobre lo que quiera añadir información?

¡Muchas gracias por su tiempo y por sus respuestas son de gran ayuda para el estudio!

Annex 2. Interview guide for healthcare workers (in Spanish)

GUÍA ENTREVISTA A MUJERES EMBARAZADAS

1. Presentación

· Buenos tardes. Lo primero, presentarme. Soy __, trabajo en el Instituto de Salud Global de Barcelona.
· En primer lugar, agradecerle su disponibilidad para participar en esta entrevista a partir de la que esperamos conocer su opinión y puntos de vista, ya que es importantes para el estudio.
· En esta entrevista vamos a hablar sobre su experiencia del embarazo y sobre su punto de vista sobre la vacunación.
· Este encuentro va a durar aproximadamente sobre media hora. Va a ser grabado con su consentimiento para asegurar que la información no se pierda. Los datos serán tratados de manera anónima y confidencial. En el momento de analizar y publicar los datos, se hablarán de las opiniones generales de todos los participantes del estudio. TODA LA INFORMACIÓN ESTARÁ CODIFICADA.

1.1. Datos sociodemográficos
Primero le voy a preguntar unos datos sociodemográficos que voy a anotar, pero que no voy a grabar. Le voy a avisar en el momento que empiece a grabar.
(DATOS SOCIODEMOGRÁFICOS)

1.2. Introducción
¡Empezamos a grabar!
· Bueno, hoy es __(fecha)__, a las__(hora)__ y esta entrevista está siendo grabada a partir de una llamada telefónica.
· Únicamente voy a recoger su consentimiento para participar de manera oral, así que, puede decir en voz alta si acepta participar en el estudio, y ser grabada.

2. Historia personal

Hábleme un poco sobre usted…

- ¿Cómo se enteró de que estaba embarazada?

- ¿Llevaba mucho tiempo buscando?
- ¿Cómo es su día a día con el embarazo? (condiciones: vida cotidiana)
- ¿Quién la ayuda? ¿Qué apoyos tiene durante el embarazo?
- ¿Cómo se siente con el embarazo?
- ¿Y a nivel de salud? ¿Tiene alguna patología?

- ¿Actualmente está trabajando?
- ¿En qué área? ¿Desde cuándo está en este lugar?
- ¿Tiene estudios en algún ámbito?

3. Percepciones generales de las vacunas
- ¿Qué opinión tiene sobre las vacunas en general? ¿Cuándo oye "vacuna", que le viene a la cabeza?
- ¿Ve igual si se trata de vacunas en personas adultas, mayores, niños/as…?
- (si no lo ha dicho ella) ¿Cree que las vacunas en general funcionan /son eficaces?

4. Percepciones generales de las vacunas en el embarazo
- ¿Y qué opina de las vacunas durante el embarazo?
- ¿Cree que las vacunas son seguras en el embarazo o que tienen algún riesgo?
- (En el caso que crea que haya riesgos) ¿Cuáles?
- (en el caso que no lo haya dicho ella) ¿Son mayores los beneficios o los riesgos? ¿Cree que en general los beneficios de las vacunas superan sus riesgos?
- ¿Cree que otras mujeres embarazadas de su entorno sienten/perciben lo mismo?

5. Conocimientos de vacunas en el embarazo
- ¿Qué sabe acerca de las vacunas en el embarazo? (tipos, administración…)
- ¿En qué momento del embarazo (semanas, trimestre…) se ponen?
- ¿Sabe que enfermedades previenen?
- ¿Para qué se utilizan? ¿Para proteger a la madre, al feto o ambos?
- ¿Cómo afecta a las mujeres embarazadas por el hecho de estar embarazadas?
- ¿Cómo cree que deciden vacunarse las mujeres embarazadas de su entorno o no hacerlo?
- ¿Van a casa y se lo piensan? (o ya lo tienen claro…)
- ¿Cree que suelen confiar en las recomendaciones de los sanitarios?
- ¿Qué opinión tiene de la vacuna de la tos ferina?
- ¿Y de la vacuna de la gripe?
- ¿Y de la vacuna de covid-19?
- ¿Cree que la gente/la sociedad tiene una percepción similar de estas tres vacunas o que hay alguna diferencia?

6. Experiencia en embarazos previos
- ¿Ha tenido otros embarazos anteriormente?
- ¿Ha sido vacunada alguna vez durante el embarazo?
- ¿De qué vacunas?
- ¿Cómo se sintió cuando se vacunó? ¿Tuvo miedo?
- En el caso de que se vacunara…
- (únicamente si ha tenido embarazos anteriores y se ha vacunado) ¿Cree que ha prevenido infecciones en su bebé?
- - En el caso de que no se vacunara…
- ¿Cuál fue el motivo? ¿Sintió miedo de hacerlo?
- ¿Quién tomó la decisión de vacunarse o no?
- ¿Cómo la tomó?
- ¿Con quién lo consultó?
- ¿Dónde se informó?

(Fuentes de información: pareja, matrona, ginecóloga, pediatra de sus otros bebes, familia, amigas, otros medios etc.)
- (Si ha tenido un hijo/a en un país extranjero), ¿conoce el calendario vacunal?

7. Experiencia en el embarazo actual
- ¿Le han propuesto vacunarse?
- ¿Y qué ha decidido? / (si es un embarazo reciente) ¿Y si se lo propusieran?
- ¿Cómo ha tomado esta decisión? / ¿Cómo tomaría esta decisión?
- ¿Cuándo le propusieron vacunarse? ¿tuvo (suficiente) tiempo para pensárselo? ¿Lo pensó mucho? ¿Por qué?
- ¿Cómo se siente/se ha sentido al tomar esta decisión?
- ¿Quién ha sido su mayor apoyo para decidir esto?
- ¿Quién tomó la decisión de vacunarse o no?
- ¿Cómo la tomó?
- ¿Con quién lo consultó?
- ¿Dónde se informó?

(Fuentes de información: pareja, matrona, ginecóloga, pediatra de sus otros bebes, familia, amigas, otros medios etc.)

8. Fuentes de confianza y credibilidad
- ¿Cuáles son sus fuentes más confiables para obtener información de las vacunas en el embarazo?
- ¿Y para decidir vacunarse?
- ¿Cree que la opinión del profesional sanitario puede afectar a su decisión de vacunarse?
- ¿Cuál cree que es el papel de las autoridades sanitarias/médicas para vacunarse/en todo esto de la vacunación?
- ¿Cree que este papel ha cambiado tras la pandemia?
- ¿Confía más o menos en las recomendaciones de vacunas?

- ¿Considera que ha tenido la opción de elegir?
- ¿Se siente que ha sido bien informada?
- ¿Qué le ha gustado de cómo se lo han contado informado y qué no?
- ¿Qué le ofrece confianza?
- ¿Qué vacuna le genera más confianza/credibilidad para vacunarse en el embarazo: tos ferina, gripe, covid y otras?
- ¿Por qué? ¿Y la que menos? ¿Por qué?

9. Ensayo clínico
- ¿Estaría dispuesta a participar en un ensayo clínico de una vacuna?
- ¿Por qué motivos?
- ¿Y en una situación de pandemia?
- ¿Conoce alguna vacuna que esté en ensayos clínicos actualmente en mujeres embarazadas?
- ¿Sabe que actualmente se están haciendo ensayos con estreptococo tipo B y virus respiratorio?
- ¿Participaría en alguno de estos ensayos?
- ¿En qué casos?
- ¿Hay alguna situación en la que cree que participaría?

10. Otros temas
- ¿Hay algún tema que no hayamos tratado y que quiera comentar o algo sobre lo que quiera añadir información?

¡Muchas gracias por su tiempo y por sus respuestas son de gran ayuda para el estudio!

Table S1. Decision-making process for vaccination during pregnancy.

Decision	Personal opinion of healthcare professionals	Information shared during consultations	Relationship between healthcare professionals and patients	Translated quotes in English	Quotes in original language (Spanish or Catalan)
Yes	IN FAVOR: Healthcare professionals in favour of vaccination.	DETAILED: The pros and cons of vaccination are explained in the consultation.	GOOD: There is a bond of trust between healthcare	"If the patient and the healthcare professional have a bond of trust and there is a good doctor-patient relationship, or between the midwife and the patient or between the gynaecologist and the patient,	"Si el paciente si el personal sanitario tiene un vínculo de confianza y hay una buena relación médico paciente o de la matrona con el paciente o del ginecólogo con el paciente, pues el paciente se va a sentir confiado y va a confiar en

			professionals and patients.	then the patient will feel confident and will trust what is in what tells you more about your gynaecologist or your midwife than another person who rarely sees you and has little relationship with the patient." 109-01	lo que en lo que le diga más de su ginecólogo o su matrona que otra persona que lo ve poco y tiene poca relación con el paciente." 109-01
Maybe	DOUBT: Doubts about the recommendation of the vaccine.	INCOMPLETE: Explains only the pros, gives little information, or shows little knowledge.	NON-EXISTENT: There is no established relationship.	"If I asked my doctor for information and saw that he had doubts, or was hesitant about some kind of message, right? Of course, it would create doubts. For me he is the person of reference" 214-01	"Si yo pidiera información a mi médico de referencia y viera que tiene dudas, o estuviera dubitativo en algún tipo de en el mensaje, ¿no? Pues claro, me crearía dudas. Para mí es la persona de referencia" 214-01
No	DOUBT OR DENIAL: Doubts about the recommendation of the vaccine.	INCOMPLETE: It is not shown with the knowledge of having sufficient scientific basis.	NON-EXISTENT: There is no established relationship.	"That is, well, at the time of vaccination, you tell him "look, vaccination of this is recommended in this trimester for this reason". And the majority… I mean, I think so, I think that if I told them "no, it's not contraindicated" or "it's not recommended", they wouldn't put it on. Or even saying "it is recommended, but I prefer that you do not do it", is that I would not do it." 105-02	"O sea, pues el momento de la vacunación, tú le dices "mira, está recomendado en este trimestre la vacunación de esto por este motivo". Y la mayoría… o sea, yo creo que sí, yo creo que si yo le dijese "no, no está contraindicado" o "no se recomienda", no se lo pondrían. O incluso diciendo "se recomienda, pero yo prefiero que no lo hagas", es que no lo haría." 105-02
No	WITHOUT IMPORTANCE: The patient already made the decision at home.	WITHOUT IMPORTANCE: The patient has the information she needs and with unshakable arguments.	NON-EXISTENT: There is no established relationship.	"Well, it also depends on how it is placed, how the information is put on it. But for example, it didn't affect me. In that sense, if you already have your ideas clear or you know... (...) And I had in mind... no, no, no, I don't think it conditions you, yes, if you know what you want or You know that you are going to inform yourself if or if from other sources it is also like one more opinion, right?" 105-01	"Bueno, depende también de como viene puesto, como se le pone la información. Pero por ejemplo, a mi no me afectó. En ese sentido, si tu ya tienes tus ideas claras o sabes... (...) Y yo tenía en mente... no, no, no, no creo que te condiciona, si, si tu sabes lo que quieres o sabes que te vas a informar si o si de otras fuentes también es como una opinión más, no?" 105-01

Table S2. Factors for decision-making and women's profiles.

Reason	Translated quotes in English	Quotes in original language (Spanish or Catalan)
Varied profile descriptions according to different factors.	"As for here, what is facing the patients I observe that there are different tendencies. It goes from the patient who is going to do what the doctor tells her and I say doctor, I do not say midwife, "what the doctor says and amen" (...) And on the other hand we have very well-informed women, who are they are the ones who are going to ask him "hey, and don't you vaccinate me for pertussis? Because I'm almost 28 weeks". In other words, there are both aspects. (...) Regarding other types of people, well, there are people who don't, who are very critical and when they go to a doctor, they are only doing a complete X-ray. (...) And that one doesn't marry anyone. She is investigating. And then there is the lady who thinks that this is a normal, physiological process and that the pharmacologists are very involved in all this and that they even have a home birth.(...) So there is everything. I think we have a good population, like fashion of all types. (...) I think it is a lack of culture, medical culture, I mean, and personal care" 202-02	"En cuanto a aquí, lo que es de cara a las pacientes yo observo que hay diferentes tendencias. Hay desde la paciente que va a hacer lo que le diga el médico y he dicho el médico, no digo comadrona, "lo que diga el médico y y amén" (...) Y por otro lado tenemos mujeres muy bien informadas, que son ellas las que van a solicitarle "oye, ¿y no me vacunas de la tosferina? Porque yo ya casi estoy de 28 semanas". O sea, hay las dos vertientes. (...) Respecto al a otro tipo de gente, pues hay personas que no, que son muy críticas y cuando llegan a un médico solo le están haciendo una radiografía total.(...) Y ese no se casa con nadie. Esta va investigando. Y luego hay la señora que piensa que esto es un proceso normal, fisiológico y que las farmacológicas están muy metidas en todo esto y que ellas tienen hasta un parto en casa.(...) Entonces hay de todo. Yo creo que tenemos una población bueno, como la moda de todos los tipos. (...) Yo creo que es falta de cultura, cultura médica, me refiero, y de cuidado personal" 202-02
About the "more naturist" profile.	"The truth is that normally the women who do not want to be vaccinated are... Well, people who want everything to be very natural... And normally it is people, the truth is that it is people from here who have everything very natural. In general, people from South American or Asian countries, Muslims, usually do accept vaccines without problems." 104-02	"La verdad es que normalmente la mujer que no se quiere vacunar es... Bueno, gente que quiere como todo muy natural... Y normalmente es gente, la verdad es que es gente de aquí que lo tiene todo como muy natural. En general, la gente de países sudamericanos o asiáticos, musulmanes normalmente sí que aceptan las vacunas sin problemas." 104-02
The study factor as a variable.	"Because sometimes people with less culture are sometimes more malleable or sometimes the other way around. Sometimes they are the ones who are more empathetic than not... and at the high cultural levels, well, in general they also understand well what you tell them, but maybe I would say that the most... for example, the anti-vaccine groups perhaps they would be more within the high socio-cultural levels." 102-02	"Perquè a vegades la gent amb menys cultura a vegades és més moldejable o a vegades al revés. A vegades són els que estan més empatiants en que no... i i als nivells culturals alts doncs bé, en general també entenen bé el que tu els hi dius, però potser jo diria que els més... per exemple, els grups antivacunes potser estarien més dintre dels nivells socioculturals alts." 102-02

The factor of origin as a variable	"However, there are cultural groups, especially those with a language barrier, who are a little more suspicious because a very different type of medicine is practiced here than what can be practiced in their society of origin. And then there are culturally rooted social groups here, right? that maybe yes they are people who ask themselves maybe more questions and maybe they are reading more and have more doubts about not only vaccines, but the medicalization of childbirth, the taking of drugs, the need to do screening tests or tests diagnostics... (...) The two groups are perhaps the most reluctant: some because they don't fit in with our system because they come from a very different social and cultural background to ours, which doesn't fit them so well and they are a little suspicious; and the others that do have western roots so to speak, don't they? that they have grown up here and that they are starting to ask some questions and consider me..." 209-02	"Però sí que hi han grups culturals sobretot amb barrera idiomàtica que desconfien una mica més perquè aquí es fa un tipus de medicina molt diferent de la que es pot fer a la seva societat d'origen. I després hi ha grups de socio culturalment arrelats aquí, no? que potser sí que són gent que es fa potser més preguntes i potser estan llegint més i tenen més dubtes respecte no només les vacunes, eh a la medicalització del part, a la presa de fàrmacs, a la necessitat de fer proves de cribatge o proves diagnòstiques… (...) Son els dos grups potser més reacis: unes per desencaix amb el sistema nostre perquè perquè vénen d'una formació social i cultural molt diferent a la nostra, que no els hi encaixa tant i desconfien una mica; i els altres que sí que són d'arrel occidental per dir-ho d'alguna manera, no? que sí que han crescut aquí i que es comencen a fer algunes preguntes i a plantejar-me..." 209-02

Table S3. Motivational factors for deciding to be vaccinated during pregnancy (including original quotes)

Reasons for accepting vaccination in pregnancy	Quotes in original language (Spanish or Catalan)
Good for the baby and the mother.	"La ginecóloga me comentó sobre la vacuna. Ehh me explico muy bien. Dijo qué beneficios tendría, para qué era buena ehh que ayudaría mi bebé, que generaría a mi cuerpo más anticuerpos. O sea, no tuve que pensarlo mucho ni que sí, ni que no. O sea, realmente estuve de acuerdo con la vacunación." 213-01
Avoiding infection.	"Entiendo que sí, que todo puede tener un riesgo, pero entiendo que son bajos respecto al riesgo que puede ser la enfermedad en sí." 204-01
Good for the community.	"En primer lugar la del COVID-19, porque creo que estamos en una situación de pandemia grave y que es inminente la necesidad, no? de frenarlo" (...) Entonces, creo que si por ejemplo te puedes vacunar y evitarlo, pues es importante. Y si con la vacuna no lo evitas al menos quizás disminuyes los efectos ehh del virus para que no sea tan violento." 203-01
Recommendation by healthcare professionals.	"Ah, es que no, o sea, para mí no es... o sea, ni me lo he planteado un rechazo, o sea, no vacunarme para mi era una vacuna vacunarme sí o sí. O sea, no era... no es discutible. (...) si un facultativo me dice que me tengo que tomar esto, pues creo que él es el que tiene criterio como para que, para que me tome un medicamento o una vacuna o lo que sea." 214-01

Trust in the healthcare system.	"... creo que depende bastante de tener tu opinión en general de la confianza que te produce el sistema sanitario y la confianza que te producen las vacunas, los medicamentos, los tratamientos, bueno, todas estas cosas." 204-01

Table S4. Motivational factors for deciding not to be vaccinated during pregnancy (including original quotes).

Reasons for refusing vaccination in pregnancy	Quotes in original language (Spanish or Catalan)
Fear that it could affect foetal development.	"Hi ha gent molt desconfiada i molt molt suspicaç. Però sí que és veritat que les dones embarassades potser evidentment tenen por de que les vacunes els hi puguin afectar al desenvolupament del fetus i que i que no vegin aquestes avantatges, no? de protegir se de totes les infeccions que poden tenir que els altres poden passar el fetus per la per la via placentària." 207-02
Low perception of disease risk.	"Porque consideraba que no... no, no... vacunarme... no tenía porque ahorrarme pasar la gripe. Y que si pasaba la gripe, pues bueno, me estaría una semana en cama con mocos. Pero bueno, que tampoco... no pasa nada, no? (...) más que no le veía la necesidad, la utilidad." 203-01
Distrust in the healthcare system.	"Els seus arguments són molt irracionals, "que si es pensen que estem aquí per ser conillets d'índies", "que si això no... no no tenim cap mena de confiança". Vull dir, de fet, jo qualsevol cosa, "jo vaig conèixer una persona que el van vacunar i mira què li va passar..." (...) No escolten i estan fan una generalització o fan una visió molt catastròfica o fan una visió molt desconfiada." 207-02
Not being able to counteract the side effects with medication.	"Hombre, yo creo... bueno, a ver, lo que sí que puede afectar un poco más es de que si te encuentras mal por así decirlo, o te coge fiebre, a lo mejor como no tienes tantos medicamentos o no tienes... sí que te puedes tomar un paracetamol. Pero bueno, tampoco es muy adecuado que te lo tomes. Pues a lo mejor pasar esos síntomas sin nada es lo que puede causar un poco de reparo" 205-01
Political views/Moral values.	"Diuen que les vacunes no són segures o que és una manipulació de les farmacèutiques. (...) I que i al final sigui que també no veuen un benefici individual, no? Com que si ha de ser un benefici grupal doncs no ho veuen" 205-02
Not believing in vaccines or other medications.	"Generalmente la gente que no se quiere vacunar tampoco quiere otras medicaciones, o sea, si son diabéticos tampoco se pincharía la insulina. O sea, suele ser gente que ya es reticente a cualquier medicación por el miedo de que…" 105-02
Fear of contracting an illness through vaccination.	"[Algú] es va posar un dia la vacuna de la grip i es pensen que va produir la grip, que no és veritat perquè no la pot produir perquè és una vacuna inactivada. Però... però bueno, és la creença que tenen. Llavors aquesta gent per més reticents a posar-se la vacuna." 102-02

Table S5. Factors for being vaccinated against pertussis during pregnancy.

Reason	Translated quotes in English	Quotes in original language (Spanish or Catalan)
Protection from a greater risk.	“Well, it seems like a useful vaccine to me. I have a friend who did not vaccinate her children and then they had the disease and it is not pleasant at all. So, pretty much in favour of avoiding that if you can. And it is a simple and low-risk way through a vaccine, I think it is a very, very good idea.” 204-01	“Pues me parece una una vacuna útil. Yo tengo alguna amiga que no vacunó a sus hijos y luego tuvieron la enfermedad y no es nada agradable. Así que, bastante a favor de evitar eso si se puede. Y es una forma sencilla y con bajo riesgo mediante una vacuna, me parece muy muy buena idea.” 204-01
Protecting the baby.	“Yes, it is true that I notice that pregnant women are more confident about pertussis because they know that it is specifically for the baby, for the newborn's immunity. So it's like I think that's why the administration gives them more confidence.” 203-02	“Si que es verdad que noto a las embarazadas como más confiadas con respecto a la de la tosferina porque saben que es específicamente para el bebé, para la inmunidad del recién nacido. Entonces es como que creo que por eso les da más confianza la administración.” 203-02
Long term experience.	"In this one, there is another part, as there is the tetanus part, so in this one... since it had been more than 10 or 12 years or 15 since I had the tetanus one, so they recommend that they put it on me.” 103-01	“En aquesta, hi ha una part més, com que hi ha la part del tètanus, doncs en aquesta sí que... com que feia més de 10 o 12 anys o 15 que m'havia pres la del tetanus, doncs em van recomanar que me la posessin.” 103-01
Uncertainty: combining pertussis with diphtheria and tetanus.	“Well, the other day there was something that... surprised me when they gave me the pertussis vaccine, they told me that it wasn't made by itself, but that it already came with tetanus and diphtheria. And of course, it's weird, isn't it? (...) I thought: "How strange that we have to give ourselves an extra dose of diphtheria, of tetanus when I think I have a dose that I think the next one will be when I'm sixty years old", but well, that's the only thing I thought. It's not very well planned, is it? It is not necessary, I do not think it is necessary to give a pregnant woman diphtheria and tetanus.” 101-01	“Bueno, el otro día hubo una cosa que me... extrañó cuando me pusieron la vacuna de la tos ferina que me dijeron que, que no se fabricaba sola, sino que ya iba con tétanos y con difteria. Y claro, es raro ¿no? (...) pensé: "Qué raro que tengamos que ponernos un extra de difteria, de tétanos cuando creo que llevo una dosis que creo que la siguiente me toca cuando tenga sesenta años", pero bueno, es lo único que pensé. No está muy bien planificado, ¿no? no hace falta, creo tampoco ponerle a una embarazada difteria y tétanos.” 101-01

Table S6. Factors for being vaccinated against influenza during pregnancy.

Reason	Translated quotes in English	Quotes in original language (Spanish or Catalan)
Perception of low risk.	The flu vaccine honestly seemed super, superfluous to me. (...) I am not an expert, I am not a doctor, but normally, luckily, I am always in good health." 105-01	"La vacuna de la gripe sinceramente me me pareció súper, superflua (...) No soy una experta, no soy un médico, pero yo normalmente, por suerte, siempre tengo siempre buena salud." 105-01
Preferring to pass the disease than have side effects.	"The one with the flu, yes it is true that I feel that there are more women who do not want it because they do not... or they have never had the flu or they have heard that it was a lot of reaction and it is like they are not afraid of catch the flu and prefer not to put more things in the body." 203-02	"La de la gripe, sí que es verdad que siento que hay más mujeres que no se la quieren poner porque no... o no ha cogido nunca la gripe o han escuchado que era mucha reacción y es como que no le no miedo a coger una gripe y prefiere no meterse más cosas en el cuerpo." 203-02
Perception of being less effective and not avoiding contagion.	"And my parents, when they have been vaccinated against the flu, the truth is that they have not been more protected. Or the feeling, as I don't understand much, is that they have caught a cold and the cold has been strong when they have been vaccinated. I mean, no, it doesn't feel like like super super protection. (...) My experience is that it hasn't been of much use to my environment today either. (...) Older people do die of the flu, many, even if they are vaccinated." 102-01	"Y mis padres cuando se han vacunado de la gripe, la verdad es que no han estado más protegidos. O el sentimiento, como no entiendo mucho, es que se han resfriado y ha sido fuerte el resfriado cuando se han vacunado. Es decir, no, no lo siento como como super super protección. (...) La experiencia que tengo es que tampoco es que haya servido a día de hoy mucho para mi entorno. (...) Las personas mayores si que mueren de gripe, muchas, aunque estén vacunadas." 102-01
Perception that its effectiveness is variable due to virus mutation.	"Sometimes it can create a little less confidence in me that I know that the flu is a virus that mutates every winter and that it can, and that there is a probability that I will get a vaccine that is not the variant or the virus that you can catch. In other words, I understand that therefore the effectiveness that I could catch the flu may be a little lower, but that there may be an equal possibility to the fact that it is not the same type of virus that I can catch." 214-01	"A veces me puede crear un poco menos de confianza el hecho de que sé que la gripe es un virus que muta a cada cada invierno y que puede, y que hay una probabilidad de que me ponga una vacuna que no sea la variante o el virus que que que pueda coger. O sea, entiendo que por lo tanto la efectividad de que yo pudiera coger la gripe puede ser un poco más baja, pero que puede haber una posibilidad igual al hecho que no sea pues el mismo tipo de virus que pueda coger." 214-01

Table S7. Factors for being vaccinated against COVID-19 during pregnancy.

Reason	Translated quotes in English	Quotes in original language (Spanish or Catalan)
Vaccination reluctance, distrust and uncertainty.	"The truth is that the one with which I have had the most reluctance has been with the COVID-19. (...) But with the COVID-19 vaccine, it has been a feeling of good, of mistrust, right? sometimes feeling a little well well like not knowing, right? Although they are telling us that it is recommended, but to what extent, right? there is enough evidence that the vaccine is safe, right? Because it seems to me that no, it doesn't cause malformations, that it doesn't cause problems, but we don't know in the future either, right? How can it affect them? And then this created a lot of mistrust in them and most consider that they preferred to wait once the baby is born and put it on more, more safely." 201-02	"La verdad es que con la que más reticencias he tenido ha sido con la del COVID-19. (...) Pero con la vacuna del COVID-19 si que ha sido una sensación de bueno, de como de desconfianza, no? de de a veces de sentirse un poco pues bueno no saber, no? Aunque nos están diciendo que si que recomendable, pero hasta que punto, no? hay suficiente evidencia de que la vacuna es segura, no? Porque me parece que no, no causa malformaciones, que no causa problemas, pero tampoco sabemos en un futuro, no? como puede afectarles, no? Y entonces esto les creaba mucha desconfianza y la mayoría consideran que preferían esperarse una vez nazca el bebé y ya ponérsela de manera más, más segura." 201-02
New vaccine and the unknown effects in the medium-long term.	"It came out very recently. And I have doubts being pregnant and not being pregnant. (...) Well, I think that in the end they have not had enough years to test this vaccine. (...) it is not possible to know the secondary effects that we are going to have in the long run. So yeah, it's a little scary because of that." 205-01	"Ha salido hace muy poco. Y me da reparo estando embarazada y no estándolo. (...) Bueno, pienso que al final no han tenido los años suficiente para testar esta vacuna. (...) no se puede llegar a saber los efectos secundarios que vamos a tener a la larga. Entonces sí que da un poco de miedo por eso." 205-01
See themselves as experimental subjects.	The COVID-19 therefore has little baggage... it has little distance. We are a bit of guinea pigs. In other words, no one says what effects the vaccine will have in five years, because basically five years ago this vaccine did not exist." 103-01	"La COVID-19 doncs té poc bag... té poc recorregut. Som una mica conillets d'índies. És a dir, ningú diu quins efectes tindrà la vacuna d'aquí a cinc anys pues perquè bàsicament fa cinc anys no existia aquesta vacuna." 103-01
Its production has been very fast.	"Yes, I think it is perceived differently because it happened very quickly. Then there is a point that on the one hand, people are aware of COVID-19 and want to protect themselves from COVID-19. (...) The fact that a vaccine has been found or sought... has been made so quickly, people also have a point of distrust." 205-02	"Si, jo crec que es percep diferent perquè s'ha produït d'una manera molt ràpida. Llavors hi ha un punt que per una banda, la gent està com conscienciada amb la COVID-19 i es vol protegir de la COVID-19. (...) El fet que s'hagi trobat o s'hagi busc... s'hagi fet una vacuna tan ràpid, la gent també hi ha un punt que desconfia." 205-02

Changes in the protocols and lack of unanimity in the recommendation.	"That on the one hand they recommended one thing, on the other hand another, right? Put it, for example, with the third dose, right? Well, better a month better than catching COVID-19, right? Or after five months, then eight months, right? I think all this information and all these contradictory messages end up creating a bit of insecurity and not knowing what is correct." 201-02	"Que por un lado recomendaban una cosa, por otro lado otra, no? Ponerla, por ejemplo, con la tercera dosis, no? Pues mejor al al mes mejor de pasar el COVID-19, no? O después a los cinco meses, luego a los ocho meses, no? Toda esta información y todos estos mensajes contradictorios yo creo que acaban creando un poco de de inseguridad y de no saber qué es lo correcto." 201-02
Its production has been made from a new technology.	"It is different from the other vaccines, it is a… it is RNA. And the other vaccines were with virus substances or live viruses. The concept is different and I believe that there is not enough long-term experience, which is surely good, but I don't know... no... there is no long-term experience." 101-02	"Es diferente a las otras vacunas, es un... es ARN. Y las otras vacunas eran con con sustancias del virus o virus vivos. Es diferente el concepto y yo creo que no hay experiencia a largo plazo suficiente, que seguramente es buena, pero pero no sé... no... no hay experiencia a largo plazo." 101-02
It does not prevent infection.	"The COVID-19 one, I admit that maybe I was more reluctant. Basically because even if you put it on many times, you end up taking it." 103-01	"La del COVID-19 jo admeto que potser era més reticent. Bàsicament perquè és que encara que te la posis moltes vegades acabes agafant-lo." 103-01
Perception of lower risk of the disease than the vaccine.	"It's not like it scares me or anything like that, but it doesn't seem as serious to me as pertussis, for the same reason a bit, because it's the disease that we were talking about the flu... right? I mean, I think not, that it doesn't have as much impact then, well, it's like more..." 204-01	"No es como que me da miedo o algo así, pero tampoco me parece igual de grave que la tos ferina, por lo mismo un poco, porque es la enfermedad que lo que comentábamos de la gripe... no? o sea, creo que no, que no tenga tanto impacto entonces bueno, es como más..." 204-01
Criticism of "widespread vaccination".	"I had COVID-19 without being pregnant. I'm supposed to have antibodies. Supposedly, huh? I don't know, no one has checked, they haven't even asked me. Hey, do you want to look at it first? No, they have decided that I have to get vaccinated just because, because it is the best, period. But my case has not been studied. So for me, I consider myself strong in terms of immunity because I went through COVID-19 and because I had a hard time, but I got through it. So I think for my case there are more risks than benefits." 102-01	"Yo pasé el COVID-19 sin estar embarazada. Se supone que yo tendría anticuerpos. Se supone, eh? no lo sé, nadie me lo ha mirado, ni siquiera me han preguntado ¿Oye, tú lo quieres mirar antes? No, ellos han decidido que me tengo que vacunar porque sí, porque es lo mejor y punto. Pero no se han estudiado mi caso. Entonces para mí, que yo me considero fuerte en tema inmune porque pasé el COVID-19 y porque lo pasé fuerte, pero lo pasé. Entonces creo que para mi caso hay más riesgos que beneficios." 102-01
Get vaccinated for the common good.	"Well, I think it is very important because at the moment we are in now it is important to stop the virus, right? and perhaps if in 10 or 20 years it were put in a systematic way, it would have to be assessed, but currently it is essential." 203-01	"Bueno, creo que es muy importante porque en el momento en que estamos ahora es importante frenar el virus, ¿no? y quizás si dentro de 10 o 20 años se pusiera de manera sistematizada, que habría que valorarlo, pero actualmente es fundamental." 203-01

Distrust of the health and/or political system.	"The truth is that I also feel that there is, as I have told you, there is a business behind all this. So we will never know the reality, or at least in the short term. So I can't know the risks because no, I'm not doing the study or I'm not doing or I'm not looking at studies, ehh. I know that if there is a pregnancy there is no risk and that the mother is immunologically strong, etc. I believe that today's COVID-19 vaccine, today's COVID-19, if there is no risk, no...no one knows, then it should not be administered in a crazy way." 207-01	"La verdad es que también siento que hay como te he dicho, hay un negocio detrás de todo esto. Entonces nunca vamos a saber la realidad, o al menos a corto plazo. Entonces los riesgos yo no lo puedo saber porque no, no hago el estudio o no estoy haciendo o no estoy viendo estudios, ehh. Sé que si hay un hay un embarazo que no es de riesgo y que la madre es fuerte immunológicamente mente hablando, etc. yo creo que la vacuna a día de hoy del COVID-19, a día de hoy del COVID-19, si no hay riesgo, no... noo se sabe, entonces no debería administrarse a lo loco." 207-01
Perceptions of complications in pregnancy.	"I perceive it differently because of the miscarriage I had. (...). Ehh but to me, I already told you, what scares me about COVID-19 is during pregnancy, after pregnancy or before pregnancy, I really have no fear of it." 106-01	"Yo la percibo diferente por el aborto que tuve. (...). Ehh pero a mi, ya le digo, la del COVID-19 lo que me da miedo es durante el embarazo, después del embarazo o antes de un embarazo, yo realmente no le tengo ningún miedo." 106-01
Exceptional situation: the pandemic.	"Distrustful people come in here, don't they? They says "yes man, a vaccine that has been there with so little experience that it has been done in a hurry." (...) but also those who may think, it's a bit like saying "of course, we are dealing with a pandemic that has never had a disease with the mortality that COVID-19 had, right? From flus from the great flu pandemics that have also occurred. But let them remember this generation of ours, one by one, how we have been, I think that justified the fact that the vaccine had to be done quickly, that it had to be imposed fast and because we were facing an emergency and a pandemic. And so much so that it was perceived, that is, it was perceived differently. But the pandemic was also different." 207-02	"La gent desconfiada entren aquí, no? Diu "si home, una vacuna que hi ha hagut amb tan poca experiència que s'ha fet de pressa i corrents". (...) però també els que poden pensar, és una mica de dir "clar, és que estem davant d'una pandèmia que mai una malaltia havia tingut la mortalitat que havia tingut el COVID-19, no? Des de les grips des de les grans pandèmies de grips que també hi han hagut. Però però que recordin aquesta generació nostra un un com hem estat, jo crec que això justificava pues que la vacuna s'ha hagut de fer de pressa, que s'ha hagut d'imposar ràpida i perquè estàvem davant d'una urgència i d'una pandèmia. I tant que s'ha percebut, o sigui s'ha percebut diferent. Però és que la pandèmia ha sigut diferent també." 207-02

Table S8. Recommendations for COVID-19 vaccine during pregnancy.

Topic	Translated quotes in English	Quotes in original language (Spanish or Catalan)
The beginnings: uncertainty and feeling of insufficient scientific basis to recommend the vaccine.	"We feel quite overwhelmed, I think, and quite insecure in the sense that it was information that varied over time and in a short time (…) And then I also experienced it a bit with a bit of overwhelm because without having a lot of information about it, more than a couple of lines in a very broad protocol, ehh they specified you briefly, the women asked a lot. (...) There was a lot of insecurity, they asked us for our opinion or advice as professionals and we could read them two lines, a protocol. Not that we had much more information." 208-02	"Nos sentimos bastante desbordadas creo y bastante inseguras en el sentido de que fue una información que fue variando a lo largo del tiempo y en poco tiempo (…) Y luego también lo viví un poco con un poco de agobio porque sin tener muchísima muchísima información al respecto, más que un par de líneas en un protocolo muy amplio, ehh te especificaban brevemente, las mujeres preguntaban mucho. (...) Había mucha inseguridad, nos pedían opinión a nosotros o consejo como profesionales y podíamos leerle dos líneas, un protocolo. No es que tuviéramos mucha más información." 208-02
Feelings of anguish, overwhelm and insecurity.	"The truth is that very bad, very bad. First because before May, I had a lot of queries, emails, calls regarding... as it was totally contraindicated, patients asked me that they could be pregnant and what they were going to do ehh if they had been vaccinated and such (...) And then suddenly from one week to the next it became highly recommended and the consultation was the other way around, that is, from patients who had doubts about putting it on and instead the guides said yes, it was recommended. But well, mmm no, we haven't had any kind of evidence. Simply the recommendation, the recommendation of the hospital or of or of or of the Generalitat. (...) The truth is, at first it was contraindicated. After, from week 20. After any time. Then the disorders that caused the period began to appear. Ehhh anyway, the truth is that with the COVID-19 vaccine it's all quite a lot... I don't know. No, it has not convinced me at all." 101-02	"La verdad es que muy mal, muy mal. Primero porque antes de mayo, tuve un montón de de consultas, correos, llamadas respecto a... como estaba totalmente contraindicada, me preguntaban las pacientes que podían estar embarazadas y que iban a hacer ehh si se habían vacunado y tal (...) Y luego de buenas a primeras de una semana para otra pasó a estar recomendadisima y la consulta fue al revés, o sea, de pacientes que tenían dudas respecto a ponérsela y en cambio las las las guías decían que sí, que era recomendable. Pero bueno, mmm no, no hemos tenido ningún tipo de evidencia. Sencillamente la recomendación, la recomendación de del hospital o de o de o de la Generalitat. (...) La verdad, al principio era contraindicado. Después, a partir de la semana 20. Después en cualquier momento. Luego empezaron a salir los trastornos que producían la regla. Ehhh en fin, la verdad es que con la vacuna del COVID-19 es todo bastante... No sé. No, a mí no me ha convencido en absoluto." 101-02
Wait for time to pass to gain confidence.	"Well, it has depended a bit on the time. Not much at first, right? because it was... well, I had your information, but still in all the studies it stated that there was a lack of evidence. So, in the end, you told the women what you really knew was "look, I have that information, but you have to be aware that so much evidence is also needed", right? that	"Pues ha dependido un poco de la época. Al principio no mucho, no? porque era... bueno, tenía su información, pero aún en todos los estudios ponía que faltaba evidencia. Entonces, al final decías a las mujeres lo que realmente sabías que era "mira, yo tengo esa información, pero hay que ser conscientes de que también hacen falta tanta evidencia", no? que ellos también lo supieran, no? Yo no me sentía

	they also knew, right? I didn't feel 100% safe recommending something if I wasn't 100% safe either.” 201-02	segura recomendando algo al 100% si tampoco estaba 100% yo segura.” 201-02
Uncertainty about side effects, lack of clinical trials, and dissatisfaction with the reporting system.	" Because there is no study, there is no data, there is nothing. (...) I think pregnant women have been and are being vaccinated without knowing what is going on. We will know, because so many have been vaccinated that the numbers are good once we collect all the side effects, we will know something. Now I also tell you that the side effects of women are not being reported because they are having many alterations with their menstrual cycles and they are not being picked up as a possible adverse effect to the vaccine.” 209-02	“Perquè no hi ha cap estudi, no hi ha cap dada, no hi ha cap res. (...) Jo crec que s'han estat i s'estan vacunant a les dones embarassades sense sense saber què passa. Ho sabrem, eh perquè s'han vacunat a tantes que so fan bé els números d'una vegada recollim bé tots els efectes secundaris, sabrem alguna cosa. Ara també et dic que no s'estan reportant els efectes secundaris de les dones perquè estan tenint moltes alteracions amb els cicles de la regla i no s'estan recollint com a possible efecte advers a la vacuna.” 209-02
Act according to unclear guidelines.	“Well, sometimes yes, but a little ambiguous maybe, right? (...) well, everything is very concise, right? Very... It was a bit of a leap of faith, wasn't it? Briefly. And where, of course, like everything, it varied from one moment to another, because it was that of saying "Oh! with what certainty can you tell someone, no?" 208-02	“Bueno, a veces sí, pero un poco ambiguas tal vez, no? (...) bueno, muy escueto todo, no? Muy... Era un poco acto de fe, no? Escuetamente. Y donde, claro, como todo, variaba de un momento a otro, pues era aquello de decir "¡Ay! ¿con qué seguridad se le puede decir a alguien, no?" 208-02
Recommend the vaccine because the institution where they work recommends it.	“I have recommended it because... because well, because I had one... what I said, because I had one... over time, yes, you feel more comfortable, right? Sure, in the beginning it is difficult. (...) There was a site written in the Catalan Health Institute that said X, well I said it. But as with other vaccines, such as the pertussis vaccine, it was very easy, very easy to find information with evidence for many years, right? Well, with the COVID-19 it was not so simple. So my recommendation was based on my institutional structure, well, good. And that's it. I repeated it like a parrot, the ICS recommendation. There it was no longer mine, it was that of the ICS.” 204-02	“La he he recomendado porque.. porque bueno, porque tenía una... lo que lo que he dicho, porque tenía una... a lo largo del tiempo ya sí, te vas sintiendo más cómoda, no? Claro, en el inicio que es difícil. (...) Había un sitio escrito en el Institut Català de la Salud que decía X, pues yo lo decía. Pero así como en otras vacunas, como la vacuna de la tosferina era muy fácil muy fácil encontrar información con evidencia de muchos años, no? Pues con la del COVID-19 no era tan sencillo. Entonces mi recomendación se basaba en que mi estructura institucional, pues bueno. Y ya está. Yo lo repetía como un loro, la recomendación del ICS. Ahí ya no era tanto la mía, era la del ICS.” 204-02
Avoid recommending the COVID-19 vaccine.	“No, discourage it, no. But hey, just be more reluctant to talk about it if the woman didn't consult." 204-02	“No, desrecomendarla, no. Pero bueno, igual ser más reticentes a a hablar de ello si la mujer no consultaba.” 204-02

Internal conflict about the recommendation.	"Well, thank God, now that the third dose has passed, more or less everyone is immunized. Well, right now, at this moment, I don't have this conflict. But I've had it, huh." 101-02	"Bueno, gracias a Dios, como ahora han pasado ya lo de la tercera dosis, que más o menos ya todo el mundo está inmunizado. Pues ahora mismo, en este momento, pues no tengo este conflicto. Pero lo he tenido, eh." 101-02
Prefer to recommend other ways of preventing the disease.	"But if I have to recommend it, during pregnancy I normally advise, if possible, that they isolate themselves and do not get vaccinated until they have the baby. (...) Well, uh, they ask about my opinion and I tell them to wait a bit. Nor do I tell them not to not get vaccinated, but to wait until the third trimester of pregnancy or after having the baby." 101-02	"Pero de tener que recomendarlo, yo en el embarazo normalmente aconsejo, si es posible, que se aíslen y no se vacunen hasta que tengan al bebé. (...) Bueno, eh, ellas preguntan en mi opinión y yo les digo que esperen un poco. Tampoco les digo que no sea que no se vacunen, sino que esperen al tercer trimestre del embarazo o después de tener al bebé." 101-02
COVID-19 vaccine recommendation. Protocol vs. reality.	"Well, at first I had... I was a little... I mean, a little more reticent. What happens is that, of course, they have made so many recommendations to us and apparently, well, it seems so. It is the only thing that I always advise them is yes, that they get vaccinated. The only thing that I do not advise is that they be very close to delivery and not the first few weeks. I always tell them well, well after the second trimester, for example, and up to 34." 103-02	"Bueno, yo yo al principio tenía... era un poco... o sea, un poco más reticente. Lo que pasa es que, claro, nos han hecho tantas recomendaciones y al parecer,bueno, parece que sí. Yo es lo único que les yo siempre lo que les aconsejo es que sí, que se vacunen. Lo único que yo no les aconsejo que sean muy cerca del parto y tampoco las primeras semanas. Yo siempre les digo bueno, pues después de la en el segundo trimestre, por ejemplo, y hasta hasta el 34." 103-02
Responsibility in the entities or institutions in charge of carrying out and supporting protocols and guidelines.	"In the case of pregnancy, they are protocols. Well, there is the protocol for the COVID-19 vaccine that is updated 12,000 times and the protocol for the pregnancy follow-ups (...) But really the reason why you recommend it is because you... let's say your institution has a protocol that recommend it. Not so much because you have read or not read, but apart from that you can do it or not, eh (...) But the reason why I recommended something is because there is a protocol that... indicates to do it and besides it safeguards me to do it. (...) for example, the COVID-19 vaccine, what I was telling you about, they no longer name it in the consultation, unless there is a direct question from the patient." 204-02	"En el caso del embarazo son protocolos. Pues hay el protocolo de la vacuna del COVID-19 que es actualizado 12.000 veces y el protocolo del seguiment de l'embaràs (...) Pero realmente el motivo por el que la recomiendas es porque tu... digamos tu institución tiene un protocolo que lo recomienda. No tanto porque tu hayas leído o no leído, pero eso a parte lo podrás hacer o no, eh (...) Pero el motivo por el que yo recomendé algo es porque hay un protocolo que me... que me lo que me indica hacerlo y aparte me salvaguarda hacerlo. (...) por ejemplo la vacuna del COVID-19 lo que te comentaba, igual ya no la nombran en la consulta, a no ser que haya una pregunta directa de la paciente." 204-02

Table S9. Protocols: perception and monitoring.

Topic	Translated quotes in English	Quotes in original language (Spanish or Catalan)
Vaccine recommendations based on protocols.	“We follow protocols. In other words, the ICS has a communication source that sends the protocols and the latest vaccine recommendations. And that's what we follow." 206-02	“Seguimos protocolos. O sea, el ICS tiene una fuente de comunicación que van mandando los protocolos y las últimas recomendaciones de la vacuna. Y es lo que seguimos.” 206-02
The personal option to delve into the bibliographic search.	“Well, in public health there are some protocols that immediately inform all of us of any changes that occur. The amount of mails and information and bibliography that we receive is incredible, right? And not only the general information to read, but also then there are those mmm, those recommendations, those care guides, okay? I mean, that's it. But aside from that, of course, currently we get a lot of information, of things from different societies.” 202-02	“Bueno, en la sanidad pública hay unos protocolos que cualquier cambio que haya inmediatamente nos informan a todos. La cantidad de mails y de información y de bibliografía que recibimos es increíble, vale? Y no solamente la información general para leer, sino que además luego hay esos mmm esos esas recomendaciones, esas guías asistenciales, vale? O sea, eso está. Pero aparte de eso, pues claro, actualmente nos llegan por diferentes sociedades, cantidad de información, de cosas.” 202-02
Protocols are to be followed.	“I would say that we all give the same information, at least where I work, because in the end we all have the same information. And I think that in the end, we all assume that vaccination is optional. (...) What I think is, that it is our obligation, is to inform them correctly so that they can make a decision with evidence, right? and in a safe way.” 201-02	“Yo diría que que todas damos la misma información, por lo menos donde yo trabajo, porque al final todas tenemos la misma. Y yo creo que al final, todas partimos de que la vacunación es opcional. (...) Lo que yo creo que sí, que es nuestra obligación, es informarles de manera correcta para que puedan tomar una decisión con evidencia, ¿no? y de una manera segura.” 201-02
“Freedom” in the use of the protocol.	“I mean, ehh, generally there is like... They give you the articles on which it is based and you can use the protocol or not. No no, I mean, we generally use it because you know it's like what, that, what science says. I mean, medically correct, but, well, then you can… I mean, you can use them or not.” 105-02	“O sea, ehh, generalmente hay como... Te ponen los artículos en los cuales está basado y tú puedes utilizar el protocolo o no. No no, o sea, generalmente lo utilizamos porque sabes que es como lo, eso, lo que dice la ciencia. O sea lo médicamente correcto, pero pero bueno, luego tú puedes... o sea, puedes usarlos o no.” 105-02
The protocol as mandatory.	"No, I don't know. In other words, we with the protocol have a... well, a position based on scientific evidence. And in principle this is the recommendation.” 102-02	“No, no sé. O sigui, nosaltres amb el protocol hi ha un... bueno, un posicionament en base a l'evidència científica. I en principi aquesta és la recomanació.” 102-02
There is no room for personal positioning because "you have	“Theoretically you have to recommend what the protocols say and leave your things aside a little, your individual position, right? If a specific vaccine is recommended by the... by the Department of Health, for example, then you have to inform the woman. (...) you	“Teóricamente tú tienes que recomendar lo que dicen los protocolos y dejar un poco de lado tus cosas, tu posicionamiento individual, no? Si una vacuna determinada está recomendada por el... por el Departament de Salut, por ejemplo, pues tú tienes que informar a la mujer. (...) tienes

to report on what is objective".	have to be as neutral as possible and not... that your positioning does not influence, right? (...) In other words, it is the person who has to have the decision at hand, but we do have to give the information that exists from the scientific evidence with the greatest possible objectivity and leave subjectivity aside. In other words, we cannot tell women what we would do, but let them give them the data that is objectively available and let them decide." 208-02	que ser lo más neutra posible y no... que tu posicionamiento no influencie, no? (...) O sea que es la persona la que tiene que tener la decisión a mano, pero sí que tenemos que dar la información que existe de la evidencia científica de la con la mayor objetividad posible y dejar la subjetividad a un lado. O sea, no no podemos decirle a las mujeres lo que nosotros haríamos, sino dejar que de darles los datos que hay objetivamente y que ellas decidan." 208-02
The margin of action.	"No, it is absolutely free. Both with the patient and with us. In other words, if, for example, I were a person who did not believe in vaccines, nobody would tell me that I had to believe and I had to tell them. No. (...) They will respect me for having this position. Which to a certain extent also has its logic." 202-02	"No, es absolutamente libre. Tanto con el paciente como con nosotros. O sea, si por ejemplo yo fuera una persona que no creo las vacunas nadie me va a decir que yo tengo que creer y les tengo que decir. No. (...) Me van a respetar que yo tenga este posicionamiento. Que hasta cierto punto también tiene su lógica." 202-02
Feeling of permissiveness when following a recommendation.	"I believe that the same should be given, but it is true that I believe that, for example, it has been possible to vary when it comes to recommending the time to administer the vaccine. I mean, like for example the COVID-19, I know that they told us that the recommendation was from the woman it is recommended to administer due to age. But the same people, in her opinion, because she still recommended putting it on when the first trimester was over and so on. " 203-02	"Yo creo que se deberían dar las mismas, pero sí que es verdad que creo que, por ejemplo, ha podido variar a la hora de recomendar el momento de administrarse la vacuna. Me refiero, como por ejemplo la del COVID-19, sé que nos dijeron que la recomendación era desde que la mujer se le recomienda por edad administrar. Pero que igual personas, por su opinión, pues igual recomendaba ponerla cuando pasara el primer trimestre y demás." 203-02
Changes in the protocol and permissiveness in the recommendation.	"I decided to wait because they didn't say at that time that it was better for pregnant women to take the second dose or their first from the second trimester. So it is true that later, in August, the Generalitat, Argimon, came out here saying that we still had to get vaccinated no matter what stage of pregnancy we were in. But I didn't get vaccinated at that time, I didn't get vaccinated in the middle of August, when there was all the... all the peak of the pandemic. Everything around me is vaccinated. I decreased social interaction and… No… I waited until we got into the second trimester because I don't think there was enough data to… I don't know… it was a bit sudden to say "no, now they can be vaccinated, no matter what stage of pregnancy they are in" 101-01	"Decidí esperarme porque tampoco en esos momentos decían que mejor las embarazadas ponerse a la segunda dosis o su primera a partir del segundo trimestre. Por lo que es verdad que luego, en agosto, salió aquí la Generalitat, Argimon, diciendo que teníamos que igualmente vacunarnos estuviéramos en la fase del embarazo que estuviéramos. Pero yo en ese momento no me vacuné, no me vacuné en pleno agosto, cuando había toda la... todo el pico de la pandemia. Todo mi alrededor está vacunado. Disminuí la interacción social y... No... Esperé hasta entrar en el segundo trimestre porque creo que no había datos suficientes como para... No sé... fue un poco repentino decir "no, ahora ya se pueden vacunar, da igual la fase del embarazo en la que estén" 101-01
Diverse recommendations	"The COVID-19 told me that I could get vaccinated as much as I wanted. It is what some said, those who said others that from week 20	"El COVID-19 me decían que me podía vacunar cuanto quisiera. Es lo que decían unos, los que decían otros que a partir de la semana 20

and the weight of the decision on women.	much better because already well, there was already more security. In other words, a lot, ehh, there wasn't much sense in everything they said. Some doctors told me yes, that at any time and others that please, that it was not said but to wait for me at 20. So it is a bit absurd.” 102-01	mucho mejor porque ya bueno, ya había más seguridad. O sea que mucha, ehh no había mucho sentido a todo lo que decían. Algunos médicos me decían que sí, que en cualquier momento y otros que por favor, que no se decía pero que me esparara a la 20. Entonces es un poco absurdo.” 102-01
Risk perception: association between miscarriage and vaccination.	“For me, for me, I really tell you that I think it was because of the vaccine. When I asked my boss that I had an miscarriage, she told me "how did you get a... the COVID-19 vaccine if my sister who is pregnant has been told not to get vaccinated for any reason until after 20 weeks pregnant?" (...) I think her daughter is two months old now. In other words, it was at that moment that she and my boss told me that her sister had not yet been vaccinated because she was not yet 20 weeks old. ” 106-01	“Para mí para mí realmente se lo digo que creo que fue por la vacuna. Cuando yo le consulto a mi jefa que yo he tenido un aborto, ella me dijo "¿cómo te has practicado un un... la vacuna del COVID-19 si a mi hermana que está embarazada le han dicho que por ningún motivo se vacune hasta pasadas las 20 semanas de embarazo?".(...) La niña de ella creo que tiene dos meses ahora. O sea que es en ese momento que que ella y que mi jefa me dijo a la hermana todavía no la habían vacunado porque no estaba todavía en las 20 semanas.” 106-01

Table S10. Reasons for not participating in a clinical trial.

Reason	Translated quotes in English	Quotes in original language (Spanish or Catalan)
	**Reasons for not participating in a clinical trial**	
Uncertainty or lack of security at a time as vulnerable as pregnancy.	“It's already normal to participate in a rehearsal, isn't it? It's a bit... To put it in some way, you feel like a guinea pig, don't you? In the sense that no, there is nothing proven, right? In other words, that it could you one way or another, right? But being pregnant you end up in such a vulnerable state, not only for you, but it can harm the baby precisely because it is a rehearsal, right? I don't think many pregnant women take the risk of being participants in clinical trials, really.” 212-02	“Ya de normal participar en un ensayo, ¿no? es un poquito... Por decirlo de alguna manera te sientes conejillo de indias, ¿no? En el sentido de que no, no hay nada probado, ¿no? O sea, que te podría de una manera o de otra ¿no? Pero estando embarazada acabas en un estado tan vulnerable, no solo para ti, sino que le pueda perjudicar al bebé precisamente por ser un ensayo,no? que yo no creo que muchas embarazadas se arriesgue a ser participantes en ensayos clínicos, la verdad.” 212-02
Perception of greater risk than benefit.	“No, pregnant would not participate. (...) It's a bit selfish, but I don't think I would put my pregnancy or the baby at risk just for that, for a clinical trial. (...) For not putting the baby at risk, above all.” 214-01	“No, embarazada no participaría. (...) Es un poco egoísta, pero creo que no no pondría en riesgo mi embarazo o el bebé sólo por eso, por un ensayo clínico. (...) Por por no poner en riesgo ni al bebé, sobre todo.” 214-01

Extra efforts involved.	"Well, well, well, for example, if it required what I know, to do a lot of complementary tests or a lot of clinical follow-up, eh? (...) What do I know, if it requires me to come once a week to Barcelona, and I have to be more tired or have to do more analyzes or things that can alter my well-being, well it would surely cost me more ." 205-02	"Ja, bueno, pues per exemple, si requerís jo que sé, fer-me, moltes proves complementàries o per molt seguiment clínic ehm no? (...) Jo què sé, si requereix que haig de venir un cop per setmana a Barcelona, i haig d'anar més cansada o m'han de fer més analítiques o coses que poden alterar el meu benestar pues segurament em costaria més." 205-02
Judgment towards clinical trials while arguing that they would not be vaccinated if there is a lack of experimentation.	"Never. Out of fear simply because I don't. No, no, no, no, I don't want to. I would never be a guinea pig at all. I love my life very much for it and my body, I mean no, no, no. (...) I think that my point of view from ignorance, a slightly more vision of me, me, me, me, I'm not going to play it, from there it doesn't happen, from there it doesn't happen in reality. And that? I think it has to be studied case by case. I believe that you cannot vaccinate the whole world in a crazy way and without faith and without having a previous study of what happens to those babies that are born in the long run for a period of time of at least 3 years and for a lot of information. Yes, I don't think so, of course. What's happening? That if there is no such vaccine you could not do this study. Already. Yeah, sure, but I wouldn't, I wouldn't. Or studies could be done with another type, I don't know, I'm not saying animals, but with another type of... I don't know, I'm not a scientist, I can't know, but I think it should be studied more, more thoroughly." 102-01	"Jamás. Por el miedo simplemente porque yo no. No, no, no, no, no quiero. No sería jamás conejillo de indias para nada. Amo mucho mi vida para ello y mi cuerpo, o sea no, no, no. (...) Yo creo que que mi punto de vista desde la ignorancia, una visión un poco más de yo, de yo, yo, yo, de no me la voy a jugar, de ahí no pasa, de ahí no pasa en realidad. ¿Y qué? Creo que se tiene que estudiar caso por caso. Creo que no puedes vacunar a todo el mundo a lo loco y sin fe y sin sin tener un estudio previo de qué pasa con esos bebés que nacen a la larga durante un periodo de tiempo de 3 años mínimo y durante mucho mucha información. Si, no creo, claro. ¿Qué pasa? Que si no hay esa vacuna no podrías hacer este estudio. Ya. Sí, claro, pero yo no lo haría, no lo haría. O se podrían hacer estudios con otro tipo, no sé, no digo animales, pero con otro tipo de... No sé, yo no soy científica, no lo puedo saber, pero creo que debe estudiarse más, más a fondo." 102-01
They empathize with the need for research, but recognize greater personal fears.	"Yes, I admit that this would cost me a lot. I mean, I empathize a lot with the researchers, don't you? with all the people who are working in medical research. (...). And I'm also aware that for example, when medicine, right? of the effect of medicines on pregnant women is very little studied because I have suffered, haven't I? being pregnant But it would be very difficult for me, I think ehh to say yes to an essay, no? that is, without knowing well what the risks might be because at the end of the day, if it's still a test..." 104-01	"Sí, reconec que això me costaria molt. O sigui, empatitzo molt amb amb les investigadores, no? amb totes les persones que esteu treballant en la investigació mèdica. (...). I també sóc conscient que per exemple, quan la medicina, no? de l'efecte dels medicaments en les dones embarassades està molt poc estudiat perquè jo he patit, no? estant embarassada. Però em costaria molt, crec ehh dir que si a un assaig, no? o sigui, sense conèixer bé quins poden ser els riscos perquè al cap i a la fi si és un assaig encara..." 104-01
Professionals who would not participate while pregnant due to a lack of security.	"No, I don't think so, eh. (...) Well, because a vaccine that does not have... if I look at it from a selfish point of view, if there is no evidence, then it would be difficult for me to participate, really." 103-02	"No, creo que no, eh. (...) Bueno, porque una vacuna que no tenga... si lo miro desde el punto de vista egoísta, si no hay evidencia, pues me costaría participar, la verdad." 103-02

	"No, I would not participate. For security issues. No, I wouldn't want to... that is, with a vaccine that has already been tested and is already known to be safe, okay. But neither a clinical trial nor phase 2 nor phase 3 I think I would not participate in either." 102-02	"No, no hi participaria. Pels problemes de seguretat. No, no voldria... o sigui, amb una vacuna que ja ha estat testada i que ja se sap que és segura, vale. Però ni un assaig clínic ni fase 2 ni fase 3 crec que tampoc no hi participaria." 102-02
Health professionals recognize the difficulty in participation.	"I think it's very... it's quite difficult, right? women access. In the end it is what we said, many have the message "if it were me I don't care, but in the end I have a baby", right? And I'm worried about how it can affect you, right? The therefore the effect the possible effect of a vaccine or a medication. So I think that in a clinical trial it costs them a little more to agree to participate." 201-02	"Yo creo que es muy... es bastante difícil, no? que las mujeres accedan. Al final es lo que decíamos, muchas tienen el mensaje de "si fuera yo no me importa, pero al final tengo un bebé", no? Y y me preocupa el cómo le puede afectar, no? El el pues el efecto el posible efecto de una vacuna o de una medicación. Entonces yo creo que en un ensayo clínico les cuesta un poquito más el el aceptar participar." 201-02
**Reasons to participate in a clinical trial**		
The vaccine is recommended by healthcare professionals.	"If it were recommended by the healthcare professional, if it were one of those that enter, let's say in the recommendations package, I would have no problem, in addition to participating in a... what I would not do would be to get an extra vaccine to participate in a... .a trial group." 204-01	"Si fuese recomendada por el personal sanitario, si fuese una de las que entran, digamos en el paquete de recomendaciones no tendría problema, además de participar en un... lo que no haría sería ponerme una vacuna extra para participar en un... un grupo de ensayo." 204-01
The reasons explained to them for participating were convincing.	"Let's see, I don't know. I guess it depends on the information they told me, the reasons or the side effects they could bring. If you could not explain it to me. I wouldn't be closed to doing it, but I would have to listen carefully to what it would be. (...) And aside, I want to say that when you're pregnant you also suffer for the child who... I mean they should explain to me what this would mean, let's say. (...)" 210-02	"A veure, no ho sé. Suposo que depèn de la depèn de la informació que m'expliquessin, els motius o els efectes secundaris que poguessin comportar. Si m'ho pogués no explicar. No estaria tancada fer-ho, però hauria d'escoltar bé lo que seria. (...) I a part, vull dir que quan estàs embarassada també pateixes pel fill que... vull dir que m'ho haurien d'explicar què comportaria això diguessim. (...)" 210-02
To generate scientific knowledge.	"In general, whenever I can, I will participate because I believe it is important to generate scientific knowledge and that is why whenever... But I would not do it per se. It is not a purpose, that is, I would not get a vaccine just to participate or generate that scientific knowledge. If not, if it corresponds to my case." 204-01<br><br>"Well, because... I mean, it can contribute things to the scientific community and to the benefit of patients." 209-01	"En general, siempre que pueda participaré porque creo que es importante generar conocimiento científico y por eso siempre que... Pero no lo haría per se. No es una finalidad, o sea, no me pondría una vacuna solo para participar o generar ese conocimiento científico. Si no, si correspondiese a mi caso." 204-01<br><br>"Bueno, porque... o sea, puede aportar cosas a la comunidad científica y en beneficio de los pacientes." 209-01

For the possible benefits.	"Because since I have already tried it and that then maybe yes, if there is another one that is better, maybe I can go better to try it." 207-01	"Porque como ya lo he probado y eso entonces tal vez sí, si hay otro que sea mejor, tal vez pueda ir mejor para probarla." 207-01
Be useful to other women.	"If my experience can be used for something, then go ahead. I don't know, I guess that's what moves all women too." 101-02	"Si se puede servir mi experiencia para algo, pues pues adelante. No sé, supongo que es lo que mueve a todas a todas las mujeres también." 101-02
Compensation at the health level.	"Well, well because there are usually trade-offs when they go into a studio, because they're either offered a free 3D scan or a free DNA test, they feel more in control... So they feel more in control and they usually like that. " 101-02	"Bueno, bueno porque normalmente hay compensaciones cuando entran en un estudio, porque o bien se les ofrece una exploración 3D o un test de ADN gratuito, se siente o más control... Entonces ellas se sienten más controladas y normalmente esto les gusta." 101-02
To ensure that the effects on the foetus were null.	"Whew! It's just that I suppose I would have to be very, very sure that the effects on the babies were nil. (...) But I don't know, if they gave me a very very very reasoned argument that this type of medicine, not specifically this one, but another, of a similar type... Or of... or this type of vaccine eh, or whatever it is, it's already been tested on many women before and it didn't have any effect, and you're actually presenting me with data, right? evidence that there is virtually zero chance of it affecting the baby, then yes." 104-01	"Uff! És que suposo que hauria d'estar molt molt segura de que que els efectes sobre els nadons fossin nuls. (...) Però no sé, si em donguessin una argumentació molt molt molt raonada de que aquest tipus de medicaments no específicament aquest, però un altre, d'un tipus semblant... O de... o aquest tipus de vacuna eh, o el que sigui pues ja s'ha provat prèviament en moltes dones i no ha tingut cap efecte i em presentessin dades realment, no? evidències de que hi ha una probabilitat pràcticament nul·la de que afecti el nadó, llavors sí." 104-01
Exceptional situation: as the only way to save the foetus.	"The only thing they could tell me is that my baby is going to die yes or yes, so we can still try this because I already know that the final result would not be good, so it would not change anything for me to try something else, but I would have to be certain death for my baby or for me and try something else new, but if it wasn't like that, no" 105-01	"La única cosa que me podrían decir es que mi bebé se va a morir si o si así que igual podemos intentar con esto porque ya sé que el resultado final no sería bueno, así que no me cambiaría nada probar con otra cosa, pero tendría que ser la muerte segura ya para mi bebé o para mi y intentar una otra cosa nueva, pero si no fuese así no" 105-01
